# Supplementary material for: Comparative genomics provides new insights into the diversity, physiology, and sexuality of the only industrially exploited tremellomycete: Phaffia rhodozyma
Source: BMC Genomics. 2016 Nov 9;17:901. doi: 10.1186/s12864-016-3244-7 (PMC5103461; doi:10.1186/s12864-016-3244-7)
Supplement: Additional file 6: — List of orphan genes with links to PFAM (related to Additional file 1: Table S1). (ZIP 1428 kb) [file 12864_2016_3244_MOESM6_ESM.zip › PFAM_UNIQUE/G02436_P.html]

#                                                                            --- full sequence --- -------------- this domain -------------   hmm coord   ali coord   env coord
  
# target name        accession   tlen query name           accession   qlen   E-value  score  bias   #  of  c-Evalue  i-Evalue  score  bias  from    to  from    to  from    to  acc description of target
  
#------------------- ---------- ----- -------------------- ---------- ----- --------- ------ ----- --- --- --------- --------- ------ ----- ----- ----- ----- ----- ----- ----- ---- ---------------------
  
G02436\_P             -            371 BNIP2                PF12496.3    127     0.037   12.9   1.5   1   1   6.6e-05     0.056   12.3   1.5    31    87   272   329   148   346 0.64 - Bcl2-/adenovirus E1B nineteen kDa-interacting protein 2  
G02436\_P             -            371 CDC45                PF02724.9    622       9.2    2.8  15.6   1   1      0.15        13    2.3  15.6   127   185   272   336   253   361 0.39 - CDC45-like protein  
G02436\_P             -            371 CENP-B\_dimeris       PF09026.5    101    0.0022   16.9   9.9   1   1   3.7e-05    0.0065   15.4   9.9     5    50   268   312   265   336 0.76 - Centromere protein B dimerisation domain  
G02436\_P             -            371 Cnd2                 PF05786.9    725         3    4.9   7.7   1   1    0.0051       4.4    4.3   7.7   305   398   257   344   241   355 0.75 - Condensin complex subunit 2  
G02436\_P             -            371 CobT                 PF06213.7    282       0.2    9.4  13.1   1   1    0.0011       0.3    8.8  13.1   215   270   271   330   242   342 0.68 - Cobalamin biosynthesis protein CobT  
G02436\_P             -            371 Daxx                 PF03344.10   713      0.97    6.5  14.5   1   1     0.018       1.5    5.9  14.5   435   511   271   340   254   366 0.42 - Daxx Family  
G02436\_P             -            371 DDHD                 PF02862.12   227     0.098   11.1   4.8   1   1   0.00046      0.18   10.2   4.8    72   186   225   337   218   355 0.68 - DDHD domain  
G02436\_P             -            371 DUF2722              PF10846.3    417       1.1    6.9  11.1   1   1    0.0044       1.8    6.2  11.1   343   400   271   327   244   358 0.59 - Protein of unknown function (DUF2722)  
G02436\_P             -            371 eIF3\_subunit         PF08597.5    245       4.7    5.4  11.6   1   1     0.022       7.9    4.6  11.6    18    81   276   339   268   363 0.54 - Translation initiation factor eIF3 subunit  
G02436\_P             -            371 Hid1                 PF12722.2    895      0.15    8.4   7.1   1   1   0.00047      0.19    8.0   7.1   617   701   261   353   212   368 0.68 - High-temperature-induced dauer-formation protein  
G02436\_P             -            371 HSP90                PF00183.13   531       2.4    5.1   6.8   1   1     0.005       3.3    4.6   6.8    22    84   265   331   257   351 0.49 - Hsp90 protein  
G02436\_P             -            371 Mpp10                PF04006.7    600       7.7    3.4  10.9   1   1     0.046        11    2.9  10.9   101   160   276   323   243   359 0.44 - Mpp10 protein  
G02436\_P             -            371 Myc\_N                PF01056.13   329       3.3    5.4   6.6   1   1     0.024       5.7    4.7   6.6   202   258   259   315   246   340 0.55 - Myc amino-terminal region  
G02436\_P             -            371 Nop14                PF04147.7    840       4.1    3.9  16.6   1   1     0.082       5.6    3.4  16.6   338   392   274   334   265   368 0.45 - Nop14-like family  
G02436\_P             -            371 Nucleoplasmin        PF03066.10   149      0.36    9.0   7.8   1   1    0.0071      0.65    8.2   7.8   114   140   282   308   251   317 0.55 - Nucleoplasmin  
G02436\_P             -            371 Pox\_Ag35             PF03286.9    200       6.1    5.0   9.7   1   1     0.036        13    3.9   9.7    79   127   283   340   250   347 0.46 - Pox virus Ag35 surface protein  
G02436\_P             -            371 PRP21\_like\_P         PF12230.3    229      0.41    8.8   3.5   1   1   0.00086      0.73    8.0   3.5   106   159   281   334   245   364 0.65 - Pre-mRNA splicing factor PRP21 like protein  
G02436\_P             -            371 RNA\_pol\_3\_Rpc31      PF11705.3    233       4.4    5.8   7.4   1   1     0.023       7.3    5.1   7.4   174   207   271   302   246   324 0.51 - DNA-directed RNA polymerase III subunit Rpc31  
G02436\_P             -            371 SDA1                 PF05285.7    324       9.6    4.0  16.6   1   1      0.14        14    3.5  16.6    94   167   272   338   263   364 0.32 - SDA1  
G02436\_P             -            371 SGT1                 PF07093.6    589    0.0075   13.4   2.4   1   1     4e-05    0.0073   13.4   2.4   491   564   270   351   112   367 0.73 - SGT1 protein  
G02436\_P             -            371 Sigma70\_ner          PF04546.8    211       6.1    5.1  11.6   1   1     0.085        12    4.1  11.6    41    82   280   321   266   357 0.53 - Sigma-70, non-essential region  
G02436\_P             -            371 Sporozoite\_P67       PF05642.6    727      0.38    7.2   8.4   1   1    0.0032      0.51    6.8   8.4    99   162   273   336   229   359 0.69 - Sporozoite P67 surface antigen  
G02436\_P             -            371 Spt5\_N               PF11942.3     97       9.9    5.5  11.0   1   1     0.096        23    4.4  11.0     7    63   283   340   279   361 0.49 - Spt5 transcription elongation factor, acidic N-terminal  
G02436\_P             -            371 SPX                  PF03105.14   275       1.1    7.6   5.5   1   1    0.0086       2.3    6.6   5.5   175   227   269   321   221   366 0.58 - SPX domain  
G02436\_P             -            371 TRAP\_alpha           PF03896.11   285       7.3    4.2   7.4   1   1      0.02        11    3.6   7.4    26    83   270   327   250   342 0.55 - Translocon-associated protein (TRAP), alpha subunit  
G02436\_P             -            371 Utp14                PF04615.8    735         4    4.1  13.3   1   1     0.058       5.5    3.7  13.3   318   406   269   345   255   367 0.36 - Utp14 protein  
G02436\_P             -            371 Vfa1                 PF08432.5    182       4.1    6.0  10.5   1   1     0.028       6.8    5.3  10.5    85   150   275   339   255   366 0.59 - AAA-ATPase Vps4-associated protein 1  
G02436\_P             -            371 YL1                  PF05764.8    240       1.8    6.8  13.3   1   1     0.021       2.9    6.1  13.3    53   131   272   349   255   369 0.56 - YL1 nuclear protein  
G02436\_P             -            371 YqfQ                 PF14181.1    161       3.2    6.4   7.1   1   1    0.0045       6.7    5.4   7.1    89   147   266   326   262   360 0.59 - YqfQ-like protein
